# Reply to the letter regarding “Chronic mechanical irritation and oral squamous cell carcinoma: A systematic review and meta-analysis

**DOI:** 10.17305/bjbms.2021.6006

**Published:** 2021-12

**Authors:** Archana A Gupta, Supriya Kheur, Saranya Varadarajan, Sameena Parveen, Harisha Dewan, Yaser Ali Alhazmi, A. Thirumal Raj, Luca Testarelli, Shankargouda Patil

**Affiliations:** 1 Department of Oral Pathology and Microbiology, Dr. D. Y. Patil Dental College and Hospital, Dr. D. Y. Patil Vidyapeeth, Pune, India; 2 Department of Oral Pathology and Microbiology, Sri Venkateswara Dental College and Hospital, Chennai, India; 3 Department of Maxillofacial Surgery and Diagnostic Sciences, College of Dentistry, Jazan University, Jazan, Saudi Arabia; 4 Department of Prosthetic Dental Sciences, College of Dentistry, Jazan University, Jazan, Saudi Arabia; 5 Department of Maxillofacial Surgery and Diagnostic Sciences, Division of Oral Pathology, College of Dentistry, Jazan University, Jazan, Saudi Arabia; 6 Department of Oral and Maxillofacial Sciences, Sapienza University, University of Rome, Rome, Italy

Dear Editor,

We thank Dr. Jian Xie for the valuable inputs on our article titled “Chronic mechanical irritation and oral squamous cell carcinoma: A systematic review and meta-analysis [1].” The first concern of Dr. Xie was that we had included two studies that were based on the same population. We re-examined these articles, one was published in 2010 [2] and the other in 2017 [3] by the same group of authors. Given the significant time difference between the two articles, we did not want to presume they were from the same sample population. There is no clear evidence that they are from the same sample population.

The second and the third comment were the differences in the risk of oral cancer concerning specific populations/culture/lifestyle. We agree with this point and that a statistical analysis to avoid the confounding factors would be vital. Elimination or accounting for the confounders through statistical analysis must have been performed by the individual studies. As authors of the systematic review, we have provided the results of the studies with emphasis on the limitations including the significance of confounders and how it could induce bias in the inference. The extent of the analysis performed in a systematic review depends primarily on the extent of the data analysis performed by the included studies. Thus, the analysis performed in the systematic review was largely restricted to the limited information provided by the individual studies included in the review.
